# High genetic correlation for milk yield across Manech and Latxa dairy sheep from France and Spain

**DOI:** 10.3168/jdsc.2021-0195

**Published:** 2022-05-21

**Authors:** C.A. Garcia-Baccino, C. Pineda-Quiroga, J.M. Astruc, E. Ugarte, A. Legarra

**Affiliations:** 1 INRA, GenPhySE, Castanet-Tolosan 31320, France; 2 Departamento de Producción Animal, Facultad de Agronomía, Universidad de Buenos Aires, Buenos Aires C1417DSQ, Argentina; 3 SAS Nucleus, Le Rheu 35650, France; 4 Department of Animal Production, NEIKER-BRTA, Basque Institute of Agricultural Research and Development, Agrifood Campus of Arkaute s/n, E-01080 Arkaute, Spain; 5 Institut de l'Elevage, Castanet-Tolosan 31321, France

## Abstract

Spanish Latxa and French Manech are dairy sheep breeds that split into Blond (Latxa Cara Rubia, LCR; Manech Tête Rousse, MTR) and Black (Latxa Cara Negra of Navarre, LCN; Manech Tête Noire, MTN) strains. Exchange of genetic material (artificial insemination doses) is becoming more and more frequent across these breeds, within color, to boost both genomic precision using a larger reference population and genetic progress using a larger selection base. This exchange leads to some rams having descendance across both countries. However, additional gains can only be achieved if the selected traits are genetically similar across countries. The objective of this work was to estimate the genetic correlation across breeds for milk yield. We combine across-country, within-color records, pedigree, and marker information. The number of animals with records oscillates from 65,000 (LCN) to 544,000 (MTR), whereas the number of connecting artificial insemination rams (with more than 10 daughters in the other country) is 381 MTR rams in LCR and 58 MTN rams in LCN. Blond strains had a stronger and more extended-in-time connection. The number of genotyped rams goes from 328 (LCN) to 4,901 (MTR). The relatedness of populations was assessed by principal component analysis and F_st_ coefficients. The genetic correlation was estimated using 2 (one per color) 2-trait models (each country a trait), including all available data (records, pedigree and genotypes), by maximum profile likelihood while fixing other variance components to within-population estimates. Results showed a closer genetic relationship of Blond strains than of Black strains (F_st_: 0.01 vs. 0.05, respectively). Genetic correlation estimates for milk yield were 0.70 in both cases. Based on F_st_ distances, we expected a lower correlation for Black strains than for Blond ones if dominance or epistasis are important. Thus, we attribute the value of this correlation not being close to 1 mostly to genotype-by-environment interaction, including on-farm management and trait modeling. Regardless, the correlation of 0.7 across populations is encouraging for future joint work of Latxa and Manech breeders, including joint genetic evaluations.

## Highlights

• In these populations, there are exchanges across-country, within-color.
• The “Blond” populations are closer than the “Black” as shown by genomic statistics or ram exchanges.
• Estimated genetic correlation across countries for milk yield is 0.7.
• Across-country joint selection will be more efficient than within-country selection.

## Graphical Abstract

**Figure:**
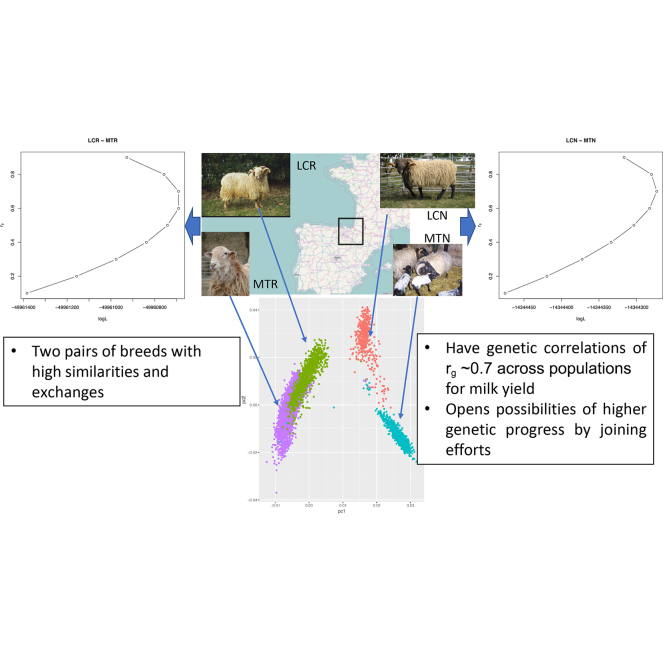
**Summary:** Latxa (from Spain) and Manech (from France) are dairy sheep breeds which split into 2 strains: Blond (Latxa Cara Rubia and Manech Tête Rousse) and Black (Latxa Cara Negra of Navarre and Manech Tête Noire). There are exchanges of genetic material and pedigree connections within strains that could make a joint evaluation and selection within strains viable. In this work we estimate the genetic correlation (r_g_) across countries for milk yield for the Blond and Black strains. In both cases the estimated genetic correlation for milk yield was 0.7. This value encourages future joint work of Latxa and Manech breeders.

Across population (or countries) genetic and genomic evaluations and later selection can lead to several benefits. Among these benefits, 3 stand out: (1) a greater genetic progress across the 2 populations (Smith and Banos, 1991), (2) a possible increase in reliability in particular for genomic predictions (Brøndum et al., 2011; Lund et al., 2011), and (3) a fairer choice of animals for breeders (Goddard, 1985).

The dairy sheep breeds Manech (in France) and Latxa (in Spain) have a common origin in the Western Pyrenees mountains and they are structured in different populations (strains or ecotypes), each with its own selection scheme (Legarra et al., 2014). Previous studies showed that pooling the data across country (within color) did not boost genomic accuracies, and also that Blond (Manech Tête Rousse, **MTR**; and Latxa Cara Rubia, **LCR**) strains are genetically very similar, and Black strains (Manech Tête Noire, **MTN**; and Latxa Cara Negra of Navarre, **LCN**) are more distinct (Legarra et al., 2014). There is nevertheless a common interest in sharing data and modifying the selection schemes toward selection across and within country. In fact, recently there had been exchanges of semen within color to explore the feasibility of joint evaluation and selection (Pineda-Quiroga et al., 2020). So, some rams have offspring, great-offspring, and so on, across both countries.

A key parameter for an across-population genetic evaluation is the correlation across breeding objectives (Smith and Banos, 1991), where a high correlation indicates that an extra genetic gain can be achieved in both populations. The correlation across countries of the individual traits per se is also of interest (Karoui et al., 2012; Wientjes et al., 2017) because even though the traits can be named identically, they can differ across countries due to genotype-genotype and genotype-environment interactions, different statistical modeling, and different trait definitions (Nilforooshan and Jorjani, 2022). A soft threshold for the joint evaluation to be of interest is an across-population genetic correlation of 0.7 (Mulder et al., 2006), although this has to be specifically analyzed for each case as it is highly dependent on the structure of the breeding scheme. Consequently, the objective of this work was to estimate the genetic correlation across countries for milk yield (**MY**), the most important trait in the breeding objective, separately for the Blond and Black strains of Latxa and Manech. If the correlation is high, exchange is useful and implementing joint evaluations will be useful too.

Data for this study came from an existing database of on-farm performance recording, and ethical approval for the use of animals was thus deemed unnecessary.

The data available for each population are described in Table 1. We combined all data available for all 4 populations' evaluation into 2 international evaluations: a Blond one (MTR and LCR) and a Black one (MTN and LCN). Most rams are AI rams, although there is some natural mating; the average number of daughters among rams with >10 daughters is roughly 150. Concerning the amount of data available (Table 1), it can readily be seen that the Blond strains are more numerous than the Black ones and that Manech has more data than Latxa. However, the quantity of data within breed is not relevant for the accuracy of across-countries correlation estimation.

**Table 1:** Description of the data used for analyses^1^

Strain	Records (MY)	Pedigree (N animals)	Rams with more than 10 daughters	Of which, genotyped rams	Animals with records for MY
MTR	1,973,609	573,501	6,432	4,901	543,929
LCR	431,692	153,765	996	716	144,993
MTN	518,226	158,055	1,233	846	146,132
LCN	197,081	68,830	459	328	65,060

1 MY = milk yield; MTR = Manech Tête Rousse; LCR = Latxa Cara Rubia; MTN = Manech Tête Noire; LCN = Latxa Cara Negra.

Given the importance of genealogical connections within color for correct estimation of genetic correlations, we also assessed pedigree completeness. Pedigree was quite complete, with 16% to 30% missing sires for the Blond strain and 25% to 32% missing sires for the Black strain. To consider missing sires, metafounders were defined every 3 yr, separately for Manech and Latxa, resulting in 10 for each within color. Pedigrees were joined within color. It was possible to do so because official identifications of Manech rams were kept when using them in Latxa.

The genealogical connection across countries is essential for accurate estimation of the correlation. Thanks to the recent exchanges, there are 381 MTR AI rams with more than 10 daughters in LCR (15% of total AI males used in LCR), and 58 MTN rams with more than 10 daughters in LCN (7% of total AI males used in LCN). Most of these rams are genotyped. These AI exchanges occurred in the vast majority (88%) after 2000 for MTR and LCR and mainly (62%) after 2005 for MTN and LCN. The exchanges only occurred in one direction: from France (Manech) to Spain (Latxa). In our files, there is no trace of a trade of live animals, which, if it occurs, is very rare.

Finally, we also used genotypes at SNP markers. Manech animals were genotyped with the 50k Illumina chip OvineSNP50, whereas Latxa animals had been genotyped with 2 chips: 50k Illumina chip OvineSNP50 and Affymetrix Axiom Ovine Genotyping Array, in proportions close to half and half (0.50 Illumina and 0.50 Affymetrix for LCR; 0.56 Illumina and 0.44 Affymetrix for LCN). Edition and quality control of the genomic information were done in national routine evaluations. We combined the 2 genotype files for a set of common markers across all 4 populations and this resulted in 22,827 SNP loci being used for the across-country estimation for both strains. The total number of animals per breed is shown in Table 1. All genotyped animals are progeny-tested AI males. The genotyped young animals without offspring were discarded because they did not provide any information.

We empirically assessed the proximity of the different populations using genomic information both visually (principal component analysis; **PCA**) and by means of the F_st_ differentiation coefficients. Then, we obtained an a priori (in the absence of data, and only due to structure and nonadditive effects) estimate of the genetic correlation, using equation (8) in Legarra et al. (2021):

$$r\approx \frac{\sqrt{h^{2}}}{\sqrt{h^{2}+\frac{8F_{st}}{1-F_{st}}c^{2}}}.$$

We used estimates of h^2^ = 0.28 and guesses of variance due to epistasis of c^2^ = 0.10.

The statistical procedure to estimate across-country correlations was as follows. First, for each color, we estimated by REML the within-country variance components using all available data within country and color, and using routine models for genetic evaluation. These models consider contemporary group, lambing season, age, and litter size at each lactation. These variance components were thus estimated with high precision as a large amount of data were available for the analysis (Table 1). We then pooled data (records, pedigree, and genotypes) within color and across country. Then, we fit a bivariate animal model where each country is a trait. We fixed the within-population variance components to these estimates. Then, we maximized the profile likelihood of the genetic correlation through a grid search from 0 to 0.9, with a step of 0.1. For each of these values of the genetic correlation, the likelihood was computed using airemlf90 (Misztal et al., 2002) using a single iterate (OPTION maxrounds 0). In this manner we obtained a profile likelihood as a function of the genetic correlation. The estimate of the genetic correlation is the peak of this likelihood, and approximate standard errors were obtained from its curvature. Note that there is no residual or permanent environment correlation because no female with a record ever belongs to the 2 populations. Because there are 2 colors, there are 2 estimates of genetic correlations across country within color (Blond and Black).

For each one of these analyses (within- and across-country estimations) we ran regular REML (with pedigrees) and single-step genomic REML (with pedigrees + markers). Results were essentially the same in both cases, so for the sake of conciseness only single-step genomic REML results are shown.

The F_st_ coefficients across MTN and LCN were 0.053, and across MTR and LCR were 0.014. This is as expected as the MTR originated from LCR in the 20th century, which is not the case for MTN and LCN, which evolved in parallel (at least for the 20th century) and also because of the larger and older use of MTR in LCR than of MTN in LCN. The other F_st_ coefficients were 0.052 (LCN − LCR), 0.061 (LCN − MTR), 0.065 (MTN − LCR), and 0.072 (MTN − MTR). The PCA in Figure 1 shows a clear overlap of MTR and LCR, as expected; indeed, due to introductions, there are LCR AI rams that are offspring of MTR AI rams. The overlap between MTN and LCN was smaller, as these breeds had less exchange, and in particular, almost no AI rams of LCN are offspring of MTN rams, although many females are.

**Figure 1:**
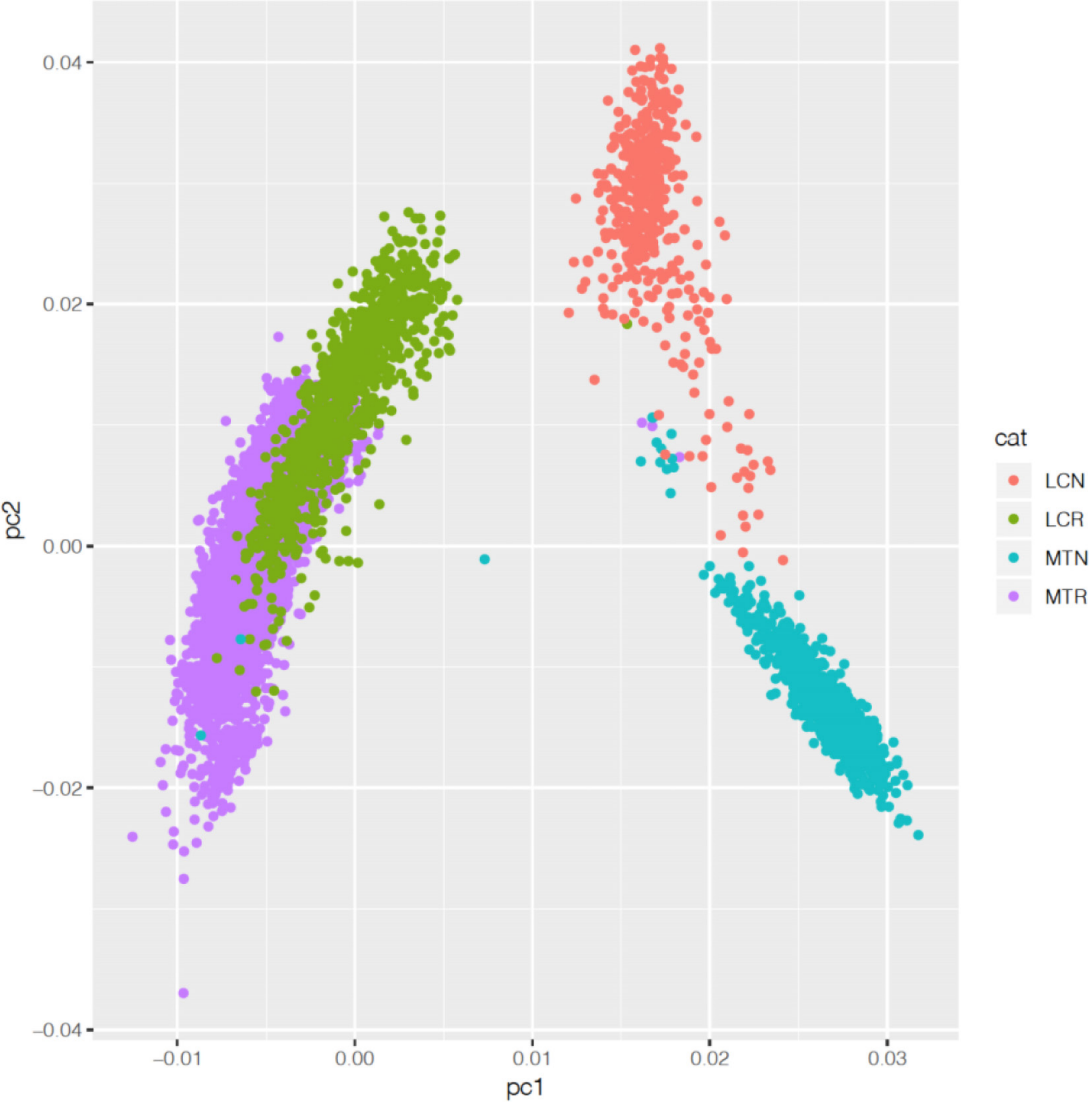
Principal components (pc) analysis showing Manech Tête Noire (MTN), Manech Tête Rousse (MTR), Latxa Cara Rubia (LCR), and Latxa Cara Negra (LCN).

Within-breed estimated heritabilities for MY were 0.28 (MTN and LCN) and 0.27 (MTR and LCR). Interestingly, repeatabilities differ, with ≈0.60 for both Manech and ≈0.40 for both Latxa. This is probably due to different treatment of first and later lactations, first due to filtering in computation of MY, and later in the respective linear models.

Figure 2 shows the profile likelihoods of genetic correlations for both LCN-MTN and LCR-MTR. In both cases the estimated genetic correlation is 0.7. Respective asymptotic standard errors were 0.05 for Black strains and 0.03 for Blond strains. The MY is computed in different ways, from lambing to 120 d in Latxa, whereas in Manech it is computed from weaning until the end of lactation, and then scaled to a constant lactation length. However, we verified that applying both definitions of MY to the same test-day data set led to a correlation of the 2 measures of MY of 0.95; consequently, this does not explain the value of 0.7.

**Figure 2:**
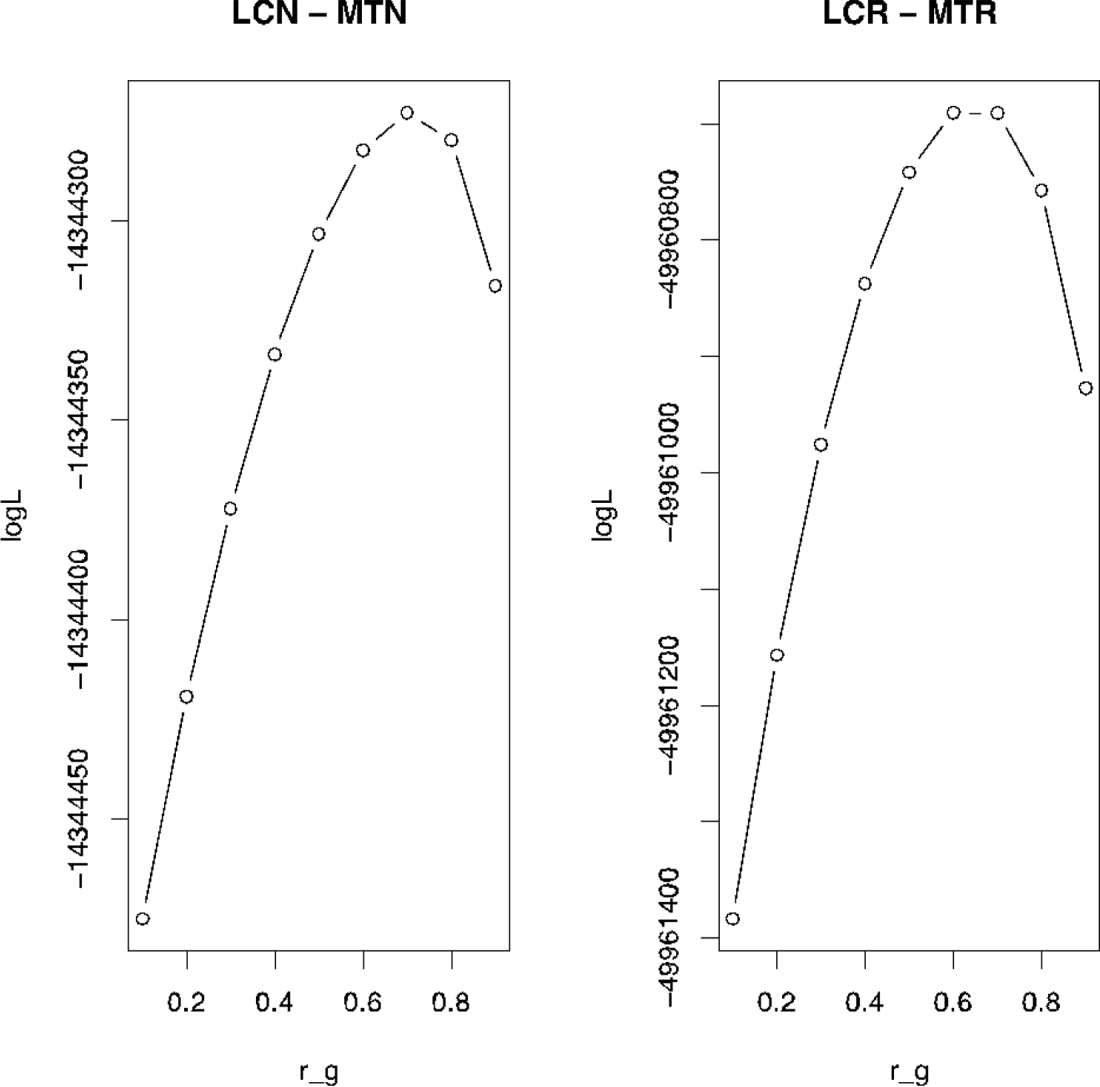
Profile log-likelihood of the genetic correlation (r_g_) for Latxa Cara Negra-Manech Tête Noire (LCN-MTN) and Latxa Cara Rubia-Manech Tête Rousse (LCR-MTR).

In addition, in presence of nonadditive genetic correlation, we expected a higher value of genetic correlation for Blond than for Black strains, because Blond populations are closer genetically as shown by the PCA in Figure 1 and by the F_st_ coefficient. The a priori estimates using F_st_ coefficients yield estimates for the correlation of 0.98 (for Blond strains) and 0.93 (for Black strains). Again, these values do not fully explain the estimate of 0.7. Thus, we speculate that most of the genetic correlation is due to genotype-environment interaction, including models used. Differences in models among countries reside in the manner of combining the elementary effects flock, age, and period in the model, and also in the effect number of lambs born, which is considered as an effect in Latxa but not in Manech. In addition, there could be some differences in farm management and climate.

Overall, the correlation of 0.7 across populations is encouraging for future joint work of Latxa and Manech breeders. New studies need to address the individual accuracy of across-population prediction, in particular for candidates to selection, and practicalities such as the optimal time frame for joint predictions (Weigel and Banos, 1997).
